# A Ride

**Published:** 1889-07

**Authors:** Loring G. Barry


### A RIDE.

We cantered down the shady road,
Princess and I,
Young, careless, wildly flirting
One Summer's day.

The glancing shafts of sunlight
Between the leaves,
Lit up her golden tresses
Like Autumn sheaves.

Dark eyes and darker lashes
Red-coral lips,
White, slender hands on bridle,
Pink finger tips.

I thought that I was flirting,
And so did she,
But when September gilds the grain
We'll wedded be.

LORING G. BARRY.

